# Effect of Pullet Vaccination on Development and Longevity of Immunity

**DOI:** 10.3390/v11020135

**Published:** 2019-02-02

**Authors:** Emily J. Aston, Brian J. Jordan, Susan M. Williams, Maricarmen García, Mark W. Jackwood

**Affiliations:** 1 Poultry Diagnostic and Research Center, Department of Population Health, College of Veterinary Medicine, University of Georgia, Athens, GA 30602, USA; ejaston@ucdavis.edu (E.J.A.); brian89@uga.edu (B.J.J.); smwillia@uga.edu (S.M.W.); mcgarcia@uga.edu (M.G.); 2 Department of Poultry Science, College of Agricultural and Environmental Sciences, University of Georgia, Athens, GA 30602, USA

**Keywords:** Infectious bronchitis virus, Newcastle disease virus, infectious laryngotracheitis virus, vaccination program, immunity, respiratory disease, ciliostasis, antibody response

## Abstract

Avian respiratory disease causes significant economic losses in commercial poultry. Because of the need to protect long-lived poultry against respiratory tract pathogens from an early age, vaccination programs for pullets typically involve serial administration of a variety of vaccines, including infectious bronchitis virus (IBV), Newcastle disease virus (NDV), and infectious laryngotracheitis virus (ILTV). Often the interval between vaccinations is only a matter of weeks, yet it is unknown whether the development of immunity and protection against challenge when vaccines are given in short succession occurs in these birds, something known as viral interference. Our objective was to determine whether serially administered, live attenuated vaccines against IBV, NDV, and ILTV influence the development and longevity of immunity and protection against challenge in long-lived birds. Based on a typical pullet vaccination program, specific-pathogen-free white leghorns were administered multiple live attenuated vaccines against IBV, NDV, and ILTV until 16 weeks of age (WOA), after which certain groups were challenged with IBV, NDV, or ILTV at 20, 24, 28, 32, and 36 WOA. Five days post-challenge, viral load, clinical signs, ciliostasis, tracheal histopathology, and antibody titers in serum and tears were evaluated. We demonstrate that pullets serially administered live attenuated vaccines against IBV, NDV, and ILTV were protected against homologous challenge with IBV, NDV, or ILTV for at least 36 weeks, and conclude that the interval between vaccinations used in this study (at least 2 weeks) did not interfere with protection. This information is important because it shows that a typical pullet vaccination program consisting of serially administered live attenuated vaccines against multiple respiratory pathogens can result in the development of protective immunity against each disease agent.

## 1. Introduction

Vaccination against respiratory viral disease is standard practice in commercial poultry operations. Both live and killed vaccines are administered to poultry, and live vaccines are commonly used for a variety of pathogens because they are effective when mass applied and are relatively economical [1]. In general, live vaccines induce local and cell-mediated immunity and provide a broader protective response than killed vaccines, whereas killed vaccines primarily induce humoral immunity and tend to be antigen-specific. The duration of immunity achieved following live vaccine administration depends on the age and type of bird, levels of maternal immunity, disease targeted by the vaccine, immunogenicity of the vaccine, method of vaccine application, number of and interval between boosters, virulence and similarity of the field challenge virus, interval between vaccination and challenge, and immunocompetency of the host [1,2,3].

Avian coronavirus infectious bronchitis virus (IBV) is an upper respiratory tract viral pathogen of poultry and leads to reduced weight gain and feed efficiency, drops in egg production and egg quality, stunted growth, and secondary bacterial infection resulting in airsacculitis [4]. The virus initially replicates in the upper respiratory tract, followed by systemic replication in the reproductive tract and some strains can cause lesions in the kidney [4]. Infected birds may exhibit nasal discharge, coughing, sneezing, and tracheal rales [4]. The disease is prevented by vaccination, and live vaccines are commonly used to induce local immunity and protection. Live vaccines are generally administered to young birds to achieve early protection, and layers and breeders are also boosted with either live or inactivated vaccines, which vary based on their similarity to the circulating field viruses [2].

Newcastle disease (ND) is caused by virulent strains of avian paramyxovirus type 1, which has recently been reclassified as avian avulavirus 1 (AAvV-1) [5]. Depending on the strain of the virus, clinical signs of ND infection may be absent or may involve depression, inappetence, respiratory signs (nasal discharge, sneezing, coughing), reduced egg production and egg quality, and neurological signs (torticollis, circling, paralysis) [3]. Strains of Newcastle disease virus (NDV) are characterized as lentogenic, mesogenic, and velogenic, according to their mean death time in embryos [6]. Lentogenic strains are of low pathogenicity causing mild respiratory or enteric infections, followed by mesogenic strains, while velogenic isolates are highly pathogenic often causing neurological signs and mortality [3]. Vaccination regimes against NDV vary and may utilize a combination of live, inactivated, and virus-vectored vaccines [7]. In the United States, the most widely used traditional vaccine strains comprise lentogenic B1 (or virus clones of the B1 strain) and LaSota strains [3].

Infectious laryngotracheitis (ILT) is a respiratory disease of poultry caused by gallid alphaherpesvirus I, and is economically important worldwide [8]. Clinical manifestations of ILT include increased mortality, reduced egg production, decreased body weight gain, conjunctivitis, tracheitis with expectoration of bloody mucus in severe cases, depression, severe dyspnea and susceptibility to other respiratory pathogens. Live vaccines against ILT virus (ILTV) may be of chicken embryo origin (CEO) or tissue culture origin (TCO), in which they are passaged multiple times in eggs or tissue culture, respectively. Although recombinant vaccines for ILT are commercially available, the CEO vaccine is the most widely used vaccine against ILTV worldwide.

Because of the need to protect chickens against different viral pathogens from an early age, vaccination programs typically include multiple vaccines against a variety of pathogens. Sample vaccination regimes in different poultry sectors are reviewed in the Merck Veterinary Manual (www.merckvetmanual.com), in which the interval between vaccinations is often only a matter of weeks. However, there is little information showing that the intervals between vaccinations are sufficient for the birds to develop adequate immune protection against challenge for each virus. The literature shows that sequential viral infections may result in viral interference, in which one virus blocks the subsequent infection and/or replication of another virus in the host [9,10], but until now it is unknown whether this phenomenon results in reduced protection from serially administered attenuated live vaccines in chickens. Interestingly, it has been reported that simultaneous administration of viruses to chickens or turkeys does not result in viral interference [11]. In this study, we investigate how a typical commercial vaccination schedule consisting of a combination of serially administered, live attenuated viral respiratory disease vaccines affects the development and longevity of immunity and protection against homologous challenge.

## 2. Materials and Methods

### 2.1. Viruses

A commercial IBV vaccine MILDVAC-GA-98^®^ (Merck Animal Health, Summit, NJ, USA) was used in this study. The vaccine was diluted according to the manufacturer’s recommendations. The challenge virus used was IBV GA98/CWL0474/98 and was prepared at a median embryo infectious dose (EID_50_) of 10^3.19^. Virus titers were calculated by the Reed and Muench method [12]. A commercial NDV vaccine B1 (NEWHATCH-C2, Merck Animal Health, Summit, NJ, USA) was used for both vaccination and challenge and was reconstituted following the manufacturer’s instructions. Since mesogenic and velogenic strains of NDV require a biosafety level above BSL2, which is not available in our laboratory, our experimental model for protection against NDV involves significantly reduced virus titers or sterile immunity against a second exposure (challenge) with the vaccine virus. An ILTV commercial CEO vaccine (Trachivax^®^ Merck Animal Health, Summit, NJ, USA) and pathogenic ILTV Georgia broiler strain 63140 [13] were used for vaccination and challenge, respectively. Strain 63140 was propagated in chicken kidney cells obtained from 3- to 4-week-old specific pathogen-free (SPF) chickens [14]. The CEO vaccine was prepared following the manufacturer’s recommendations. After inoculation, the median tissue culture infective dose (TCID_50_) was confirmed by titration of both viruses in chicken kidney cells as previously described [14].

### 2.2. Experimental Design

Specific-pathogen-free eggs were obtained at 18 days of incubation and hatched at the Poultry Diagnostic and Research Center, Athens, GA. Chicks were placed on fresh pine shavings in colony houses and pens. Chicks were vaccinated with the manufacturers recommended dose in 100 µL via the oculonasal route according to the following schedule; IBV at 1 DOA, NDV at 2 weeks of age (WOA), IBV at 4 WOA, ILTV at 8 WOA, NDV at 12 WOA, and ILTV at 16 WOA. In addition, a control group was not vaccinated. Homologous challenges were conducted at 20, 24, 28, 32, and 36 WOA, and necropsies were performed five days post-challenge (dpc). At challenge, birds received one of four treatments: IBV GA98, NDV B1, ILTV 63140, or no challenge. The treatment groups for each challenge virus per time point were as follows: non-vaccinated, non-challenged (*n* = 9–10); vaccinated, non-challenged (*n* = 9–10); vaccinated, challenged (*n* = 17–19); non-vaccinated, challenged (*n* = 9–10). All IBV-challenged birds received an EID_50_ of 1 × 10^3.2^ per bird in 100 µL intranasally. All NDV-challenged birds received the NDV B1 vaccine in 100 µL intranasally, reconstituted according to the manufacturer’s protocol. All ILTV-challenged birds received the 63140 pathogenic strain at a dose of 1 × 10^3.5^ TCID_50_ per bird in 100 µL split equally between eyedrop and intranasal routes. For IBV and NDV challenges, birds were observed at 5 dpc for respiratory signs, as previously described [15]: 0 = absent; 1 = mild; 2 = moderate; 3 = severe. For ILTV challenges, birds were observed at 3 and 5 dpc for dyspnea, conjunctivitis, depression, and mortality, as described previously [16]. The choanal cleft (IBV- and NDV-challenged and control birds at 5 dpc) or trachea (ILTV-challenged and control birds at 3 and 5 dpc) was swabbed for virus detection, and swabs were stored in PBS at −80 °C. At 28, 32, and 36 WOA, 50 µL of tears was collected by adding granulated NaCl to the eye. Blood was collected by wing or cardiac puncture and added to a microcentrifuge tube to collect serum for antibody detection. Birds were humanely euthanized, and the eyelid, Harderian gland (HG), thymus, liver, spleen, cecal tonsils, and bursa of Fabricius were collected and stored at −80 °C for virus detection and in 10% neutral buffered formalin. The trachea was removed, and one section was placed in 10% neutral buffered formalin, and the remaining portion of the trachea was submerged in tissue culture media for the ciliostasis test described below. The procedures were approved by the University of Georgia Institutional Animal Care and Use Committee (AUP #: A2015 05-001-R2).

### 2.3. Ciliostasis Test

The ciliostasis test was performed on harvested tracheas collected in cell culture media (Dulbecco’s Modified Eagle’s Medium) at 37 °C. For each trachea, five tracheal rings measuring approximately 1 mm thick were cut and represented the proximal, middle, and distal portions [17,18]. Cilia activity was observed using an inverted microscope (Olympus, Center Valley, PA, USA). The scoring system follows: 0 = all cilia beating; 1 = 75% of cilia beating; 2 = 50% of cilia beating; 3 = 25% of cilia beating; 4 = no cilia beating as previously described [17]. The maximum possible score for each trachea is 20. Each tracheal ring was scored by three individuals, and the average total score for each trachea was calculated. The ciliostasis protection score for each group was determined by the following formula: 100 − [(total of the individual scores for the group)/(the number of individuals in the group × 20) × 100], and a score ≥50 was considered protected. 

### 2.4. Tracheal Histopathology

A section of each trachea was fixed in 10% neutral buffered formalin, processed, embedded in paraffin, and 5-μm sections were cut for hematoxylin and eosin staining. For IBV lesions, epithelial hyperplasia, lymphocyte infiltration, and epithelial deciliation were scored for each trachea. Scores were determined as follows: 1 = normal, 2 = focal, 3 = multifocal, and 4 = diffuse, as described previously [19]. For NDV lesions, a descriptive analysis was performed. For ILTV lesions, microscopic lesions were scored on a scale of 0–5 (normal to very severe), as described previously [20].

### 2.5. RNA Extraction and Quantitative Reverse Transcriptase Polymerase Chain Reaction (qRT-PCR)

For IBV and NDV detection, viral RNA extraction from 50 µL of the PBS from each swab was conducted using a 5× MagMAX-96 Viral Isolation Kit (Thermo Fisher, Waltham, MA, USA) on a MagMAX™ Express-96 Deep Well Magnetic Particle Processor (Thermo Scientific, Waltham, MA, USA), according to the manufacturer’s instructions. The quantitative reverse transcription polymerase chain reaction (qRT-PCR) was performed with the AgPath-ID^TM^ One-Step RT-PCR kit (Thermo Fisher, Waltham, MA, USA), following the manufacturer’s protocol. Each 25-µL reaction mixture contained 12.5 µL of 2× RT-PCR buffer, 10 µM of each primer, 4 µM of probe, 1 µL of 25× RT-PCR enzyme mix, and 5 µL of viral RNA. The qRT-PCR reactions were run on the Applied Biosystems^®^ 7500 Fast Realtime PCR system (Life Technologies Ltd., Carlsbad, CA, USA) under the following conditions: one cycle of 50 °C for 30 min and 95 °C for 15 min, followed by 40 cycles of 94 °C for 1 s and 60 °C for 60 s. The primers and probe for the IBV qRT-PCR were previously published [21], and are comprised of a forward primer IBV5′GU391 (5′-GCT TTT GAG CCT AGC GTT-3′), a reverse primer IBV5′GL533 (5′-GCC ATG TTG TCA CTG TCT ATT G-3′), and a Taqman^®^ dual-labeled probe IBV5′G probe (5′-FAM-CAC CAC CAG AAC CTG TCA CCT C-BHQ1-3′). Primers and probe for the NDV qRT-PCR were previously described [22] and are comprised of a forward primer NDV M+4100 (5’-AGT GAT GTG CTC GGA CCT TC-3’), a reverse primer NDV M-4220 (5’-CCT GAG GAG AGG CAT TTG CTA-3’), and a Taqman^®^ dual-labeled probe NDV M+4169 (5’-FAM-TTC TCT AGC AGT GGG ACA GCC TGC-BHQ1-3’). The primers were obtained from Integrated DNA Technologies (Coralville, IA, USA), and the Taqman probe was synthesized by BioSearch Technologies (Novato, CA, USA). Real-time RT-PCR components and thermocycler parameters were previously described [21]. The data are expressed as the average cycle threshold (CT) value for all samples in each group, with positive CT values based on the limit of detection for this test associated with virus detection in eggs [23].

Each qRT-PCR reaction plate included a standard curve as an RNA extraction control and as a positive control. GA98 IBV isolated from allantoic fluid was used as the template for the standard curve. Negative controls were also included in each plate and consisted of PCR reagents with no RNA.

### 2.6. DNA Extraction

For ILTV, total DNA was extracted from the tracheal swabs using the MegaZorb^®^ DNA extraction miniprep 96-well kit (Promega, Madison, WI, USA), as described previously [24].

### 2.7. qPCR

Duplex real-time PCR assay that amplifies a fragment of the *UL44* viral gene in ILTV and a fragment of the chicken alpha 2-collagen gene was performed, as previously described [25].

### 2.8. Serum Infectious Bronchitis Virus (IBV)-Specific IgG Antibody Titers

IBV-specific IgG titers were detected using a commercial IgG enzyme-linked immunosorbent assay (ELISA) IBV antibody test kit (IDEXX, Westbrook, ME, USA). Briefly, serum samples (stored at −20 °C) were diluted 1:500, and the procedure was performed according to the manufacturer’s protocol.

### 2.9. Tear-Secreted IBV-Specific IgA Antibody Titers

Tear IBV-specific IgA was detected using a commercial IgG ELISA IBV antibody test kit (IDEXX, Westbrook, ME, USA). Briefly, tears were serially diluted two-fold in PBS and incubated in duplicate in wells overnight at 4 °C. All wash steps were performed using PBS-Tween 20 (0.05% Tween 20). Plates were incubated at 23 °C for 2 h in monoclonal mouse anti-chicken IgA-BIOT (1:1000, clone A-1, Southern Biotech, Birmingham, AL, USA), followed by 1hr in Streptavidin-HRP (1:4000, Southern Biotech, Birmingham, AL, USA). Final antibody detection steps were completed according to the manufacturer’s instructions. Endpoint titers were determined by reporting the lowest dilution at which the optical density (OD), recorded at 650 nm wavelength, was at least three standard deviations above the mean of 12 control wells incubated with no tear samples. Data from wells with a pinpoint color change due to residual substrate or air bubbles were excluded from analysis, and results were reported as log_2_ of the endpoint titer.

### 2.10. Statistical Analysis

The data were analyzed using Prism v.6.0 software (GraphPad Software, Inc., La Jolla, CA, USA; www.graphpad.com). For viral load data, a one-way analysis of variance (ANOVA) with Dunnet’s posttest was used to compare treatment groups within each collection period. All other data were analyzed using a Kruskal–Wallis test with Dunn’s posttest to compare treatment groups within each collection period. Significant differences were determined at *p* < 0.05.

## 3. Results

### 3.1. Infectious Bronchitis Virus

At 5 days following challenge with IBV GA98, vaccinated/challenged birds had significantly lower RNA loads compared to positive controls at all collection times and in all tissue samples, with the exception of cecal tonsil at 24 WOA (Table 1). In vaccinated controls, no IBV RNA was detected at all collection times except in the cecal tonsils at 20 and 24 WOA and in the choanal cleft at 20 WOA. IBV loads in negative controls in all tissues were below the limit of detection using the CT value of ≥36.17 as previously reported [23], at all collection times except the 24 WOA HG negative controls.

Clinical signs of IBV infection measured at 5 days post-challenge were significantly reduced in vaccinated/challenged birds when compared to positive controls in all weeks except 28 WOA, but trends in clinical sign scores were numerically lower among vaccinated/challenged birds (Table 2). Clinical signs were absent in non-challenged negative and vaccinated control birds, and signs in vaccinated/challenged birds were not significantly different from signs in non-challenged controls.

Histopathological examination of tracheas from all groups ranged from within normal limits to focal to multifocal minimal to moderate lymphocytic tracheitis; however, moderate lymphocytic infiltration was more frequently seen in positive controls. In all weeks, the proportion of vaccinated/challenged birds with deciliation or acute tracheal necrosis was significantly reduced compared to positive controls and was not different from negative and vaccinated controls (Table 2).

**Figure 1:**
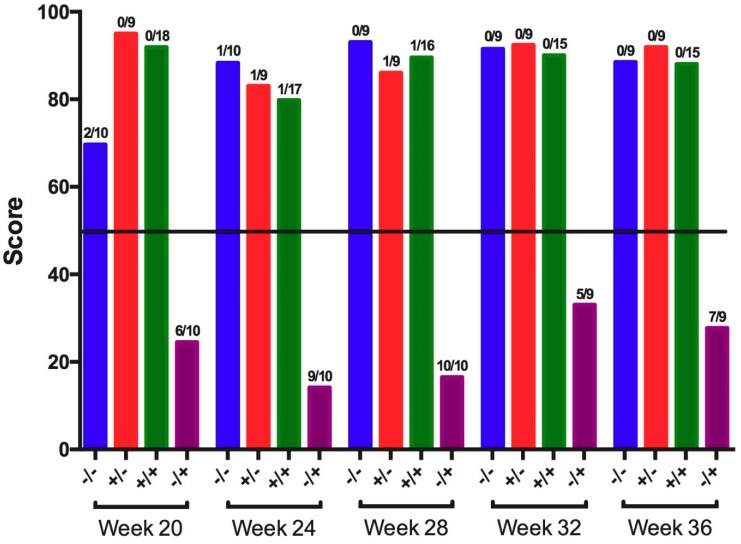
Ciliostasis protection scores among tracheas collected 5 days post-challenge with IBV GA98 for different groups of birds challenged at 20, 24, 28, 32, and 36 weeks of age (WOA). Groups with a protection score >50 (horizontal line) are protected, and groups with a protection score <50 are not protected. Fractions above each bar represent the proportion of birds positive for ciliostasis for the respective group. −/− = non-vaccinated/non-challenged, +/− = vaccinated/non-challenged, +/+ = vaccinated/challenged, −/+ = non-vaccinated/challenged.

Ciliostasis, defined as the cessation of tracheal ciliary movement, was measured at 5 dpc, and the number of birds positive for ciliostasis and ciliostasis protection scores were calculated for each group (Figure 1). At all collection times, vaccinated/challenged birds were protected from ciliostasis (scores were >50), and positive controls were not protected (scores were <50). The non-challenged negative controls and vaccinated controls were protected (scores were >50) at all collection times.

**Figure 2:**
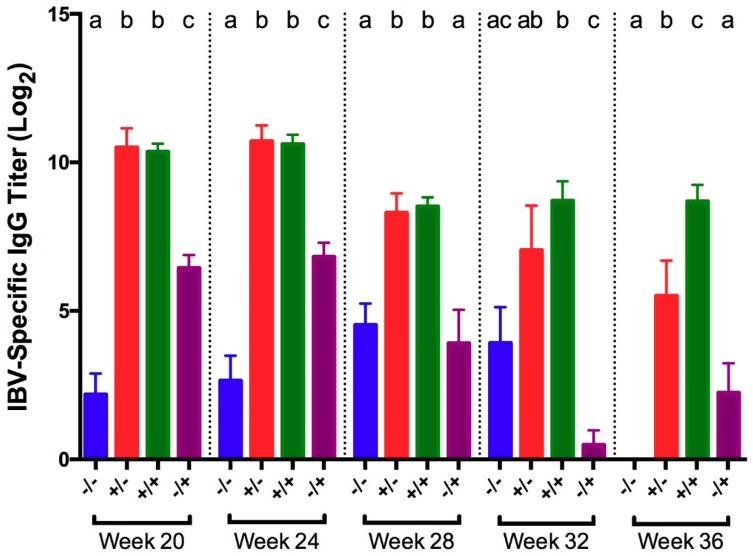
IBV-specific IgG titers in serum collected 5 days post-challenge with IBV GA98 for different groups of birds challenged at 20, 24, 28, 32, and 36 WOA. Letters (a–c) indicate significant differences among vaccine and challenge groups for each week (*p* < 0.05). −/− = non-vaccinated/non-challenged, +/− = vaccinated/non-challenged, +/+ = vaccinated/challenged, −/+ = non-vaccinated/challenged.

IBV-specific IgG titers were measured in serum collected at 5 dpc. At all times except at 32 WOA, vaccinated birds from both non-challenged and challenged groups exhibited significantly higher titers compared to non-vaccinated birds from both non-challenged and challenged groups (Figure 2). At 32 WOA, titers in vaccinated birds, regardless of challenge status, were significantly higher compared to positive controls. Titers in vaccinated/challenged birds did not significantly differ from titers in vaccinated controls until 36 WOA, when vaccinated/challenged birds had significantly higher titers. Compared to negative controls, vaccinated/challenged birds had significantly higher titers at all times.

**Figure 3:**
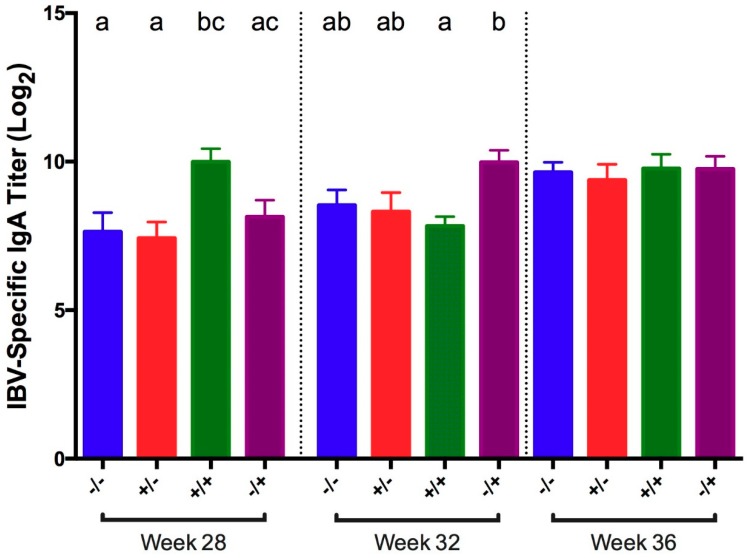
Tear IBV-specific IgA titer in tears collected 5 days post-challenge with IBV GA98 for different groups of birds challenged at 20, 24, 28, 32, and 36 WOA. Letters (a–c) indicate significant differences among vaccine and challenge groups for each week (*p* < 0.05). −/− = non-vaccinated/non-challenged, +/− = vaccinated/non-challenged, +/+ = vaccinated/challenged, −/+ = non-vaccinated/challenged.

IBV-specific IgA titers were measured in tears collected at 5 dpc at 28, 32, and 36 WOA. At 28 WOA, titers in vaccinated/challenged birds were significantly higher compared to titers in non-challenged negative controls (Figure 3). At 32 WOA, vaccinated/challenged birds showed significantly lower titers compared to positive controls. In addition, the vaccinated/challenged birds had significantly higher titers than the vaccinated/unchallenged group at 28 WOA. No other significant differences were detected.

### 3.2. Newcastle Disease Virus (NDV)

At 5 dpc with NDV B1, vaccinated/challenged birds either had undetectable RNA loads or significantly lower loads compared to positive control titers at all collection times and in all tissues sampled (Table 3). Non-challenged negative controls and vaccinated controls were negative for RNA virus using the CT value of ≥35.0 as previously reported [22].

Clinical signs measured at 5 dpc were significant at 20 WOA in positive controls, after which clinical signs in positive controls were no different from any of the other treatment groups (Table 4). No significant differences in clinical signs existed between vaccinated/challenged birds and non-challenged controls at any time. In all weeks, histopathological examination of tracheas was within normal limits or revealed focal to multifocal minimal to mild lymphocytic tracheitis, and there were no group-related differences.

Tracheas were also evaluated for ciliostasis, and no groups exhibited ciliostasis.

**Figure 4:**
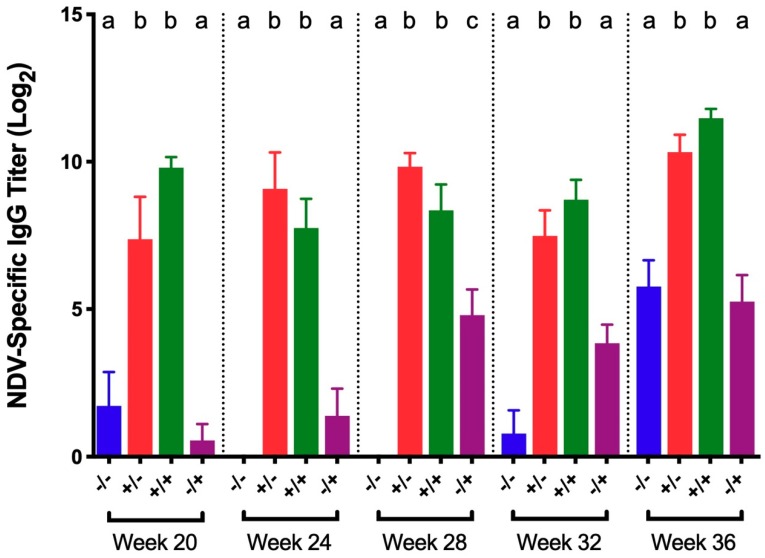
NDV-specific IgG titers in serum collected 5 days post-challenge with NDV B1 vaccine for different groups of birds challenged at 20, 24, 28, 32, and 36 WOA. Letters (a–c) indicate significant differences among vaccine and challenge groups for each week (*p* < 0.05). −/− = non-vaccinated/non-challenged, +/− = vaccinated/non-challenged, +/+ = vaccinated/challenged, −/+ = non-vaccinated/challenged.

NDV-specific serum IgG titers in vaccinated/challenged birds and vaccinated controls were significantly higher than titers in both positive and negative controls at all collection times (Figure 4). There was no significant difference in titers between vaccinated/challenged birds and vaccinated controls at any time. Similarly, no significant difference in titers was detected between non-vaccinated/challenged and negative controls, except at 28 WOA in which titers from non-vaccinated/challenged controls were significantly higher.

### 3.3. Infectious Laryngotracheitis Virus (ILTV)

At 5 dpc with ILTV strain 63140, vaccinated/challenged birds had significantly lower viral DNA loads than positive controls at all collection times and in all tissues (Table 5). The DNA loads in the trachea and HG were undetectable or low and did not differ significantly from DNA loads in non-challenged negative and vaccinated controls, which were negative using the CT value of ≥38.0 as previously reported [26]. In the conjunctiva, DNA loads in vaccinated/challenged birds were significantly higher than DNA loads from both non-challenged negative and vaccinated controls, except at 28 WOA in which no significant difference was detected between vaccinated/challenged birds and vaccinated controls.

Clinical signs and viral DNA were detected in challenged birds at 3 dpc, but signs in positive controls had become more severe by 5 dpc as tracheal viral load increased (Table 6). Clinical sign scores measured at 5 dpc were significantly reduced in vaccinated/challenged birds compared to positive controls, at all collection points except 36 WOA when clinical sign scores were numerically reduced. All negative controls and vaccinated controls had no clinical signs, and clinical signs in vaccinated/challenged birds were not significantly different compared to these controls, except at 36 WOA when clinical sign scores were significantly higher.

**Figure 5:**
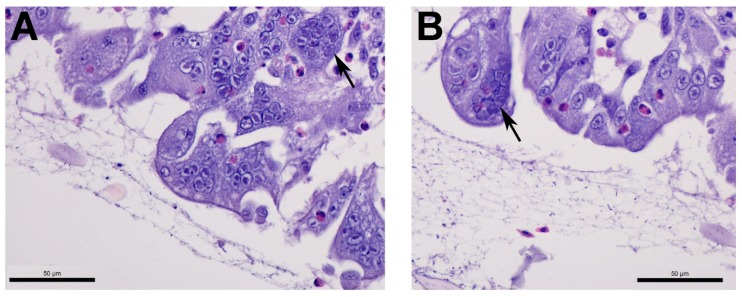
Tracheal microscopic lesions consistent with infectious laryngotracheitis infection at 20 weeks-of-age. (**A**) Numerous syncytia with intranuclear inclusions (arrow). (**B**) Another trachea from the same group demonstrating syncytia with intranuclear inclusions (arrow).

Tracheal histological examination was within normal limits or revealed focal to diffuse minimal to mild lymphocytic tracheitis in tracheas from all groups. For the positive controls, 3/9 and 1/10 in weeks 20 and 28, respectively, had acute focal necrotizing tracheitis with syncytia and intranuclear inclusions (Figure 5).

No ciliostasis was observed in the tracheas of birds from any of the groups.

**Figure 6:**
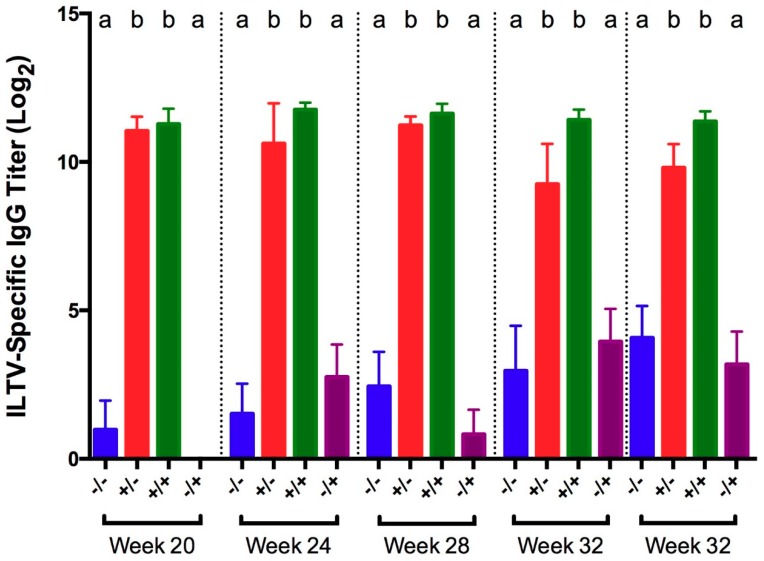
ILTV-specific IgG titers in serum collected 5 days post-challenge with ILTV strain 63140 for different groups of birds challenged at 20, 24, 28, 32, and 36 WOA. Letters (a–b) indicate significant differences among vaccine and challenge groups for each week (*p* < 0.05). −/− = non-vaccinated/non-challenged, +/− = vaccinated/non-challenged, +/+ = vaccinated/challenged, −/+ = non-vaccinated/challenged.

ILTV-specific IgG titers in serum collected 5 days post-challenge were significantly higher in vaccinated birds from both challenged and non-challenged groups, compared to the positive and negative controls (Figure 6).

## 4. Discussion

In the present study, we demonstrated that pullets serially administered live attenuated vaccines against IBV, NDV, and ILTV were protected against homologous challenge with IBV, NDV, or ILTV for at least 36 weeks, as determined by challenge virus detection, clinical signs, histopathology and ciliostasis at 5 days after challenge.

Additionally, our study showed that the age at vaccination and intervals between each vaccination did not interfere with the development of immunity to each virus and consequently protection against homologous challenge. We designed our vaccination protocol to represent a typical vaccination program for IBV, NDV, and ILTV in commercial pullets. With the knowledge that live vaccine viruses can persist in flocks, it has been unclear, until now whether the immunity induced by a live vaccines could be compromised because of viral interference, a phenomenon in which one replicating virus blocks the infection and/or replication of another virus [9,10]. Although the vaccines in the present study were administered at intervals of 2 or 4 weeks, it is feasible that virus from a previous immunization was still present at the time of the subsequent vaccination. IBV vaccines have been detected in the respiratory tract up to 28 days post-vaccination [27], and IBV was isolated from tracheal and cloacal swabs collected at the point of lay and 19 weeks of age in hens that had been virus-negative for several weeks following recovery from inoculation at one day of age [4]. Fentie, et al. [28] reported that chickens vaccinated with NDV B1 shed vaccine virus 14 days post-inoculation. In addition, a study by Hughes, et al. [29] demonstrated intermittent shedding in the trachea from ILTV-immunized chickens between 7 and 14 weeks post-vaccination. 

Few experimental studies of sequential virus infections have been published, and fewer yet have been considered in the context of poultry viral respiratory pathogens. Costa-Hurtado, et al. [30] demonstrated that chickens and turkeys serially infected with a mesogenic strain of NDV and highly pathogenic avian influenza virus (HPAIV) 3 days apart resulted in an initial decrease followed by a subsequent increase in replication of the second virus. In a subsequent study [11], the same group found that low pathogenic avian influenza virus given 3 days after a lentogenic strain of NDV did show viral interference whereas viral interference was not observed when the viruses were given simultaneously. We did not measure vaccine virus replication in the present study and, therefore, could not determine whether one vaccine virus compromised the infection and replication of a subsequent vaccine virus. However, our goal was to determine if chickens sequentially vaccinated with all three viruses were protected from viral replication and clinical signs following homologous challenge. Thus, regardless of viral interference, our data shows that immunity to individual vaccine viruses was not compromised following sequential administration of multiple live attenuated vaccines targeting different viral respiratory tract pathogens.

The detection of IBV RNA in the cecal tonsils of vaccinated/non-challenged birds at 20 and 24 WOA but not at subsequent times indicates that residual vaccine virus RNA remained in the cecal tonsils until at least 24 WOA, following the second IBV vaccination. IBV has been isolated from the cecal tonsils at 14 weeks post-infection and is known to persist for several months in various internal organs [4,31]. IBV RNA was also detected in non-challenged negative control HG at 24 WOA but was absent from all other tissues collected from negative controls, which displayed no clinical signs of IBV infection. It is not clear why we detected IBV RNA in samples from the HG in negative control birds but likely represents cross-contamination during processing of the samples since IBV was not detected in any other tissue.

The tracheal histopathological lesions of deciliation in positive controls were consistent with previous reports of IBV-induced histopathology [32,33], and were further confirmed by the presence of ciliostasis among positive controls. As expected, IBV-vaccinated/challenged birds were protected from ciliostasis. The European Pharmacopoeia states that ciliostasis can be used to evaluate IBV vaccine efficacy, in which a lack of ciliostasis would indicate that the vaccine was efficacious [34]. Therefore, these observations further confirm that IBV-vaccinated birds were protected from homologous challenge.

The observation of robust IBV-specific serum IgG titers in vaccinated birds is consistent with previous studies showing that IBV infection stimulates a humoral response in chickens [35], but that circulating antibody titers do not correlate with resistance to infection [36]. Therefore, the presence of IBV-specific serum IgG titers indicates only that the bird has been exposed to vaccine or challenge virus and should not be correlated with other measures of protection. The lack of significant titers in non-vaccinated/challenged birds can be explained by the early time of collection post-challenge. Orr-Burks, et al. [37] found that significant changes in IgG serum titer were not detected until 10 days post-inoculation.

Orr-Burks, et al. [37] also demonstrated a lack of significance in IBV-specific IgA titer in tears 5 days after both primary and secondary exposure to IBV, but IgA titer was significantly higher between 6 and 16 days after primary exposure to IBV. In this study, IBV-specific IgA titers measured in tears at 5 dpc did not reveal consistent trends and may be explained by the early time of collection post-infection. It was beyond the scope of this study to collect samples after 5 dpc, but the production of IBV-specific IgA in tears at later time points was demonstrated in a different experiment in which tears were collected between 10 and 14 dpc. In that experiment, naïve chickens challenged with IBV showed a higher trend of specific IgA titer compared to non-challenged controls [38].

The decision to use the B1 vaccine for NDV challenge was based on biosecurity regulations and the lack of appropriate biosafety level 3 facilities needed for challenge with mesogenic or velogenic strains of NDV. Since we used a lentogenic strain of NDV as challenge virus in our experimental design, protection was primarily based on significantly reduced or no virus detection at 5 days after challenge, which is a measure of local immunity preventing virus infection and/or replication. Presumably a bird protected from infection with a lentogenic strain of NDV would also show some level of protection from exposure to mesogenic or velogenic strains. Vaccinated birds at each sampling time had significantly lower or no challenge virus RNA compared to positive control groups, indicating that the vaccinated birds developed a local immune response and were indeed protected whereas the non-vaccinated positive controls were not. Because lentogenic NDV only causes a mild respiratory or enteric infection [3], it was not surprising that respiratory signs and histological changes were mild or absent despite the presence of viral RNA. We observed clinical signs of NDV infection only at 20 WOA in positive controls, while only a few birds showed mild clinical signs at 24 and 28 WOA, and no clinical signs of disease were observed at later challenge times. This observation is consistent with existing knowledge that ND tends to be more severe in younger birds [39].

The lack of ciliostasis observed in NDV B1-infected positive controls contrasts with previous reports demonstrating that NDV caused ciliostasis in tracheal explants. Butler, et al. [40] demonstrated that NDV caused ciliostasis within 2 to 6 days after infection of tracheal explants, and Malo, et al. [41] reported that following vaccination of one-day-old chicks with lentogenic NDV, the peak of ciliostasis occurred at 5 and 7 dpv and waned by 13 dpv. The discrepancy between our results and the previous studies may be explained by the use of a different lentogenic NDV strain; B1 in our studies and LaSota in previous reports [41]. It is well established that the LaSota vaccine strain is more virulent than B1 [42], which may explain the ciliostasis observed in LaSota-vaccinated chicks or LaSota-infected tracheal explants, whereas ciliostasis was not observed in our study using B1 vaccine.

Robust NDV-specific circulating IgG antibody responses developed following NDV vaccination and stayed elevated, which was consistent with prior research indicating that antibodies may be detected for up to one year in birds immunized multiple times against NDV [39]. With the exception of 28 WOA, the IgG titers in non-vaccinated/challenged birds were not significantly increased compared to titers in negative controls. This observation is not surprising given that NDV-specific antibodies are not detected in the serum until 6–10 days after exposure [39].

For the ILTV-challenged birds, vaccination prevented challenge virus replication in trachea and HG but did not completely block virus replication in the conjunctiva, especially at 32 and 36 WOA. This may suggest a waning of the local immunity against ILTV after the second vaccination at 16 WOA. In addition, the proximity of the conjunctiva to the inoculation site (eyedrop and intranasal) might explain why the local immune response was not able to completely block viral replication in the conjunctiva but successfully cleared the virus before it could replicate in HG and trachea.

Very few positive controls (non-vaccinated/challenged) demonstrated histological evidence of ILT infection in the trachea, though all positive controls tested positive for ILT DNA by PCR. This finding is not surprising in light of a report by Guy, et al. [43], which illustrated that histological detection of ILT is highly specific (98%) but poorly sensitive (42%). Notably, intranuclear inclusion bodies are present only during the initial infection (1–5 days) and disappear following epithelial cell necrosis and desquamation [44], which may explain the observation that by 5 dpc only 3/9 and 1/10 positive controls at 20 and 28 WOA, respectively, had histological evidence of ILT infection despite the presence of ILT DNA in the trachea.

The absence of ciliostasis observed in both vaccinated and non-vaccinated, ILTV-challenged birds at 5 dpc was not surprising given that Butler, et al. [40] found that only some strains of ILTV caused ciliostasis and did not correlate with virulence. Moreover, the authors showed that ciliostasis rarely occurred before 6 days, and sometimes even 9 days, after inoculation. In addition, Gerganov and Surtmadzhiev [45] also demonstrated ciliostasis in ILTV-infected tracheal organ cultures at 7–8 days post-infection. Therefore, our results combined with previous studies indicate that measuring ciliostasis may not be a reliable marker of protection from ILTV infection and that ciliostasis in ILTV infection studies may need to be evaluated at later times post-inoculation.

The lack of significant ILTV-specific serum IgG titers in non-vaccinated positive controls may be explained by the early time of collection post-challenge, as ILTV-specific antibodies do not become detectable until 5–7 dpi and peak at 21 dpi [46]. However, it is worth noting that antibody titers are not correlated to protection from ILTV infection [44]. IgG titers were robust in vaccinated birds throughout the study and support previous data that antibodies may be detectable for at least a year [44].

Taken together, our data indicate that a typical pullet vaccination program consisting of serially administered live attenuated vaccines against IBV, NDV, and ILTV does not interfere with immune responses to the individual vaccines, and the birds are adequately protected against homologous challenge until at least 36 WOA. This information is important because it shows that consecutively administered live attenuated vaccines against multiple respiratory pathogens can be an effective vaccination strategy for the development of protective immunity against each disease agent in long-lived birds.

## Figures and Tables

**Table 1:** Average quantitative reverse transcription polymerase chain reaction (qRT-PCR) cycle threshold (CT) values for infectious bronchitis virus (IBV) RNA collected from choanal cleft, Harderian gland, conjunctiva, and cecal tonsil 5 days post-challenge with IBV GA98 at 20, 24, 28, 32, and 36 weeks of age. Letters (a–d) indicate significant differences among vaccine and challenge groups for each week (*p* < 0.05).

Week	Treatment		Mean ± SEM			
	Vaccine	Challenge	Choanal Cleft	Harderian Gland	Conjunctiva	Cecal Tonsil
20	−	−	40.0 ± 0.0 ^a^	40.0 ± 0.0 ^a^	40.0 ± 0.0 ^a^	40.0 ± 0.0 ^a^
	+	−	36.5 ± 0.9 ^b^	40.0 ± 0.0 ^a^	40.0 ± 0.0 ^a^	24.9 ± 1.9 ^b^
	+	+	27.9 ± 0.8 ^c^	33.2 ± 0.6 ^b^	31.9 ± 0.7 ^b^	30.2 ± 1.2 ^c^
	−	+	17.5 ± 0.3 ^d^	26.1 ± 1.3 ^c^	22.7 ± 0.6 ^c^	21.6 ± 1.1 ^b^
24	−	−	40.0 ± 0.0 ^ab^	34.4 ± 1.0 ^a^	40.0 ± 0.0 ^a^	40.0 ± 0.0 ^a^
	+	−	41.1 ± 0.5 ^a^	39.0 ± 0.7 ^b^	40.0 ± 0.0 ^a^	30.7 ± 3.3 ^b^
	+	+	39.6 ± 0.4 ^b^	33.8 ± 0.5 ^a^	31.0 ± 0.7 ^b^	30.5 ± 1.7 ^b^
	−	+	34.3 ± 0.5 ^c^	21.0 ± 1.5 ^c^	24.1 ± 0.8 ^c^	26.1 ± 2.0 ^b^
28	−	−	40.0 ± 0.0 ^a^	39.2 ± 0.6 ^a^	40.0 ± 0.0 ^a^	40.0 ± 0.0 ^a^
	+	−	40.0 ± 0.0 ^a^	40.0 ± 0.0 ^a^	40.0 ± 0.0 ^a^	39.6 ± 0.4 ^a^
	+	+	30.4 ± 1.2 ^b^	30.9 ± 1.7 ^b^	31.5 ± 1.0 ^b^	33.7 ± 1.6 ^b^
	−	+	22.3 ± 0.3 ^c^	20.4 ± 1.3 ^c^	22.8 ± 0.5 ^c^	24.3 ± 1.8 ^c^
32	−	−	40.0 ± 0.0 ^a^	38.9 ± 0.8 ^a^	40.0 ± 0.0 ^a^	40.0 ± 0.0 ^a^
	+	−	40.0 ± 0.0 ^a^	38.6 ± 0.7 ^a^	40.0 ± 0.0 ^a^	40.0 ± 0.0 ^a^
	+	+	32.3 ± 0.5 ^b^	34.1 ± 0.4 ^b^	34.5 ± 1.6 ^b^	34.5 ± 2.0 ^a^
	−	+	20.6 ± 0.5 ^c^	21.8 ± 1.3 ^c^	24.2 ± 0.7 ^c^	23.4 ± 1.3 ^b^
36	−	−	40.0 ± 0.0 ^a^	40.0 ± 0.0 ^a^	40.0 ± 0.0 ^a^	40.0 ± 0.0 ^a^
	+	−	40.0 ± 0.0 ^a^	40.0 ± 0.0 ^a^	40.0 ± 0.0 ^a^	40.0 ± 0.0 ^a^
	+	+	30.0 ± 0.8 ^b^	30.3 ± 0.9 ^b^	28.3 ± 1.4 ^b^	32.5 ± 1.0 ^b^
	−	+	22.2 ± 0.4 ^c^	21.2 ± 1.4 ^c^	25.0 ± 0.5 ^c^	23.1 ± 1.2 ^c^

Notes: SEM = Standard error of the mean.

**Table 2:** Clinical signs and microscopic lesions measured 5 days following IBV GA98 challenge. Letters (a–b) indicate significant differences among vaccine and challenge groups for each week (*p* < 0.05).

Week	Treatment		Clinical Signs	Histopathology Lesions	
	Vaccine	Challenge	Mean ± SEM	Deciliation	Necrosis
20	−	−	0.0 ± 0.0 ^a^	2/10 ^a^	0/10 ^a^
	+	−	0.0 ± 0.0 ^a^	1/9 ^a^	1/9 ^a^
	+	+	0.0 ± 0.0 ^a^	0/18 ^a^	0/18 ^a^
	−	+	0.7 ± 0.2 ^b^	8/10 ^b^	8/10 ^b^
24	−	−	0.0 ± 0.0 ^a^	0/10 ^a^	0/10 ^a^
	+	−	0.0 ± 0.0 ^a^	0/9 ^a^	1/9 ^a^
	+	+	0.1 ± 0.1 ^a^	0/16 ^a^	0/16 ^a^
	−	+	1.0 ± 0.3 ^b^	6/8 ^b^	5/8 ^b^
28	−	−	0.0 ± 0.0	0/9 ^a^	0/9 ^a^
	+	−	0.0 ± 0.0	0/9 ^a^	0/9 ^a^
	+	+	0.1 ± 0.1	1/17 ^a^	2/17 ^a^
	−	+	0.5 ± 0.3	6/10 ^b^	6/10 ^b^
32	−	−	0.0 ± 0.0 ^a^	0/0 ^a^	0/0 ^a^
	+	−	0.0 ± 0.0 ^a^	0/9 ^a^	0/9 ^a^
	+	+	0.1 ± 0.1 ^a^	0/16 ^a^	0/16 ^a^
	−	+	1.2 ± 0.4 ^b^	7/9 ^b^	5/9 ^b^
36	−	−	0.0 ± 0.0 ^a^	0/9 ^a^	0/9 ^a^
	+	−	0.0 ± 0.0 ^a^	0/9 ^a^	0/9 ^a^
	+	+	0.1 ± 0.1 ^a^	0/16 ^a^	0/16 ^a^
	−	+	0.4 ± 0.3 ^b^	6/9 ^b^	7/9 ^b^

Notes: SEM = Standard error of the mean.

**Table 3:** Average qRT-PCR CT values for NDV RNA collected from choanal cleft, Harderian gland, and conjunctiva 5 days post-challenge with NDV B1 vaccine. Letters (a–c) indicate significant differences among vaccine and challenge groups for each week (*p* < 0.05).

Week	Treatment		Mean ± SEM		
	Vaccine	Challenge	Choanal Cleft	Harderian Gland	Conjunctiva
20	−	−	40.0 ± 0.0 ^a^	40.0 ± 0.0 ^a^	39.7 ± 0.4 ^a^
	+	−	40.0 ± 0.0 ^a^	40.0 ± 0.0 ^a^	40.0 ± 0.0 ^a^
	+	+	40.0 ± 0.0 ^a^	40.0 ± 0.0 ^a^	38.2 ± 0.6 ^a^
	−	+	29.3 ± 1.1 ^b^	28.6 ± 0.9 ^b^	27.6 ± 1.9 ^b^
24	−	−	40.0 ± 0.0 ^a^	40.0 ± 0.0 ^a^	40.0 ± 0.0 ^a^
	+	−	40.0 ± 0.0 ^a^	40.0 ± 0.0 ^a^	40.0 ± 0.0 ^a^
	+	+	40.0 ± 0.0 ^a^	40.0 ± 0.0 ^a^	39.5 ± 0.5 ^a^
	−	+	26.6 ± 1.0 ^b^	34.2 ± 1.4 ^b^	27.8 ± 1.0 ^b^
28	−	−	40.0 ± 0.0 ^a^	40.0 ± 0.0 ^a^	40.0 ± 0.0 ^a^
	+	−	40.0 ± 0.0 ^a^	40.0 ± 0.0 ^a^	40.0 ± 0.0 ^a^
	+	+	40.0 ± 0.0 ^a^	40.0 ± 0.0 ^a^	37.0 ± 0.7 ^b^
	−	+	24.5 ± 0.7 ^b^	33.0 ± 1.3 ^b^	27.9 ± 0.8 ^c^
32	−	−	40.0 ± 0.0 ^a^	40.0 ± 0.0 ^a^	40.0 ± 0.0 ^a^
	+	−	40.0 ± 0.0 ^a^	40.0 ± 0.0 ^a^	40.0 ± 0.0 ^a^
	+	+	39.2 ± 0.6 ^a^	40.0 ± 0.0 ^a^	40.0 ± 0.0 ^a^
	−	+	27.7 ± 2.2 ^b^	31.5 ± 1.6 ^b^	28.6 ± 1.2 ^b^
36	−	−	40.0 ± 0.0 ^a^	40.0 ± 0.0 ^a^	40.0 ± 0.0 ^a^
	+	−	40.0 ± 0.0 ^a^	40.0 ± 0.0 ^a^	40.0 ± 0.0 ^a^
	+	+	38.0 ± 1.1 ^a^	39.1 ± 0.7 ^a^	39.6 ± 0.4 ^a^
	−	+	24.9 ± 1.0 ^b^	33.7 ± 1.8 ^b^	30.3 ± 0.5 ^b^

Notes: SEM = Standard error of the mean.

**Table 4:** Clinical signs measured 5 days following NDV B1 challenge. Letters (a–b) indicate significant differences among vaccine and challenge groups for each week (*p* < 0.05).

Week	Treatment		Clinical Signs
	Vaccine	Challenge	Mean ± SEM
20	−	−	0.0 ± 0.0 ^a^
	+	−	0.0 ± 0.0 ^a^
	+	+	0.4 ± 0.2 ^a^
	−	+	1.6 ± 0.2 ^b^
24	−	−	0.0 ± 0.0
	+	−	0.0 ± 0.0
	+	+	0.0 ± 0.0
	−	+	0.2 ± 0.1
28	−	−	0.0 ± 0.0
	+	−	0.0 ± 0.0
	+	+	0.0 ± 0.0
	−	+	0.1 ± 0.1
32	−	−	0.0 ± 0.0
	+	−	0.0 ± 0.0
	+	+	0.0 ± 0.0
	−	+	0.0 ± 0.0
36	−	−	0.0 ± 0.0
	+	−	0.0 ± 0.0
	+	+	0.0 ± 0.0
	−	+	0.0 ± 0.0

Notes: SEM = Standard error of the mean.

**Table 5:** Average qPCR CT values for ILTV DNA collected from trachea, Harderian gland, and conjunctiva 5 days post-challenge with pathogenic ILTV strain 63140. Letters (a–c) indicate significant differences among vaccine and challenge groups for each week (*p* < 0.05).

Week	Treatment		Mean ± SEM		
	Vaccine	Challenge	Trachea	Harderian Gland	Conjunctiva
20	−	−	40.0 ± 0.0 ^a^	40.0 ± 0.0 ^a^	40.0 ± 0.0 ^a^
	+	−	40.0 ± 0.0 ^a^	40.0 ± 0.0 ^a^	40.0 ± 0.0 ^a^
	+	+	40.0 ± 0.0 ^a^	39.7 ± 0.3 ^a^	35.4 ± 1.2 ^b^
	−	+	24.2 ± 0.4 ^b^	22.7 ± 0.4 ^b^	18.2 ± 1.4 ^c^
24	−	−	40.0 ± 0.0 ^a^	40.0 ± 0.0 ^a^	40.0 ± 0.0 ^a^
	+	−	40.0 ± 0.0 ^a^	40.0 ± 0.0 ^a^	38.7 ± 0.9 ^a^
	+	+	40.0 ± 0.0 ^a^	40.0 ± 0.0 ^a^	34.5 ± 0.9 ^b^
	−	+	25.2 ± 1.1 ^b^	26.0 ± 1.4 ^b^	17.3 ± 0.8 ^c^
28	−	−	40.0 ± 0.0 ^a^	40.0 ± 0.0 ^a^	40.0 ± 0.0 ^a^
	+	−	40.0 ± 0.0 ^a^	40.0 ± 0.0 ^a^	36.8 ± 1.3 ^b^
	+	+	39.5 ± 0.3 ^a^	40.0 ± 0.0 ^a^	38.5 ± 0.6 ^ab^
	−	+	23.9 ± 0.9 ^b^	22.5 ± 1.4 ^b^	17.0 ± 0.5 ^c^
32	−	−	40.0 ± 0.0 ^a^	40.0 ± 0.0 ^a^	40.0 ± 0.0 ^a^
	+	−	40.0 ± 0.0 ^a^	39.7 ± 0.4 ^a^	40.0 ± 0.0 ^a^
	+	+	39.7 ± 0.3 ^a^	40.0 ± 0.0 ^a^	27.2 ± 0.6 ^b^
	−	+	24.7 ± 1.0 ^b^	24.2 ± 1.1 ^b^	17.7 ± 0.6 ^c^
36	−	−	40.0 ± 0.0 ^a^	40.0 ± 0.0 ^a^	40.0 ± 0.0 ^a^
	+	−	40.0 ± 0.0 ^a^	40.0 ± 0.0 ^a^	39.5 ± 0.5 ^a^
	+	+	39.8 ± 0.2 ^a^	38.3 ± 0.8 ^a^	32.0 ± 0.5 ^b^
	−	+	26.4 ± 1.7 ^b^	34.9 ± 1.6 ^b^	18.8 ± 0.9 ^c^

Notes: SEM = Standard error of the mean.

**Table 6:** Clinical signs measured 5 days following ILTV 63140 challenge. Letters (a–b) indicate significant differences among vaccine and challenge groups for each week (*p* < 0.05).

Week	Treatment		Clinical Signs
	Vaccine	Challenge	Mean ± SEM
20	−	−	0.0 ± 0.0 ^a^
	+	−	0.0 ± 0.0 ^a^
	+	+	0.6 ± 0.2 ^a^
	−	+	4.7 ± 0.7 ^b^
24	−	−	0.0 ± 0.0 ^a^
	+	−	0.0 ± 0.0 ^a^
	+	+	0.0 ± 0.0 ^a^
	−	+	2.5 ± 0.7 ^b^
28	−	−	0.0 ± 0.0 ^a^
	+	−	0.0 ± 0.0 ^a^
	+	+	0.1 ± 0.1 ^a^
	−	+	4.9 ± 0.7 ^b^
32	−	−	0.0 ± 0.0 ^a^
	+	−	0.0 ± 0.0 ^a^
	+	+	0.4 ± 0.1 ^a^
	−	+	4.0 ± 0.7 ^b^
36	−	−	0.0 ± 0.0 ^a^
	+	−	0.0 ± 0.0 ^a^
	+	+	1.1 ± 0.2 ^b^
	−	+	2.9 ± 0.4 ^b^

SEM = Standard error of the mean.
